# Preventive health counselling during antenatal care using the pregnancy risk assessment monitoring system (PRAMS) in Ireland

**DOI:** 10.1186/s12884-020-2756-y

**Published:** 2020-02-11

**Authors:** Marion Murphy, Sheena McHugh, Linda M. O’Keeffe, Richard A. Greene, Paul Corcoran, Patricia M. Kearney

**Affiliations:** 1 School of Medicine, University College Cork, Cork, Ireland; 2 Department of Epidemiology and Public Health, University College Cork, Western Gateway Building, Cork, Ireland; 3 Bristol Medical School, Oakfield House, MRC Integrative Epidemiology Unit at the University of Bristol, Oakfield Grove, Bristol, BS8 2BN UK; 4 National Perinatal Epidemiology Centre, Cork University Maternity Hospital, Wilton, Cork, Ireland

**Keywords:** Lifestyle, Pregnancy, Antenatal, Public health, women’s health

## Abstract

**Background:**

Maternal behaviours during pregnancy have short- and long-term consequences for maternal and infant health. Pregnancy is an ideal opportunity to encourage positive behaviour change. Despite this, limited information exists about the nature and content of lifestyle advice provided by healthcare professionals during antenatal care. Pregnancy Risk Assessment Monitoring System (PRAMS) Ireland is based on the Centers for Disease Control and Prevention (CDC) developed PRAMS that monitors maternal behaviours and experiences before, during and after pregnancy. The aim of the study was to assess the prevalence of preventive health counselling during pregnancy.

**Methods:**

Secondary data analysis of the PRAMS Ireland study. Using hospital discharge records, a sampling frame of 2424 mother-infant pairs was used to alternately sample 1212 women whom had recently given birth. Preventive health counselling was defined as advice during antenatal care on smoking, alcohol, infant feeding and weight gain. Self-reported maternal behaviours (smoking/alcohol cessation, gestational weight gain, infant feeding). Univariate and multivariable analyses were conducted, adjusting for maternal characteristics.

**Results:**

Among 718 women (61% response rate), the reported counselling rates were 84.8% for breastfeeding (*n* = 592), 48.4% for alcohol (*n* = 338), 47.6% for smoking (*n* = 333) and 31.5% for weight gain (*n* = 218). Women who smoked pre–pregnancy (23.7%, *n* = 170) were more likely to receive counselling on its effects compared to non-smokers (Adjusted Odds Ratio (AOR) 2.72 (95% Confidence Interval (CI), 1.84–4.02)). In contrast, women who did not breastfeed (AOR 0.74, 95%CI 0.44–1.26) and those who reported alcohol consumption pre-pregnancy (AOR 0.94, 95%CI 0.64–1.37) were not more likely to receive counselling on these topics.

**Conclusion:**

Pregnancy is an ideal opportunity to encourage positive behaviour change. Preventive health counselling during pregnancy is not routinely provided and rates vary widely depending on the health behaviour. This study suggests that additional strategies are needed to promote positive behaviour before and during the unique opportunity provided by pregnancy.

## Introduction

Maternal behaviours during pregnancy, including smoking, alcohol consumption and excessive gestational weight gain, are associated with adverse pregnancy outcomes [1, 2]. These modifiable behaviours also affect subsequent maternal health and long-term offspring health [3]. Maternal behaviours are also important at a wider societal level [4]. Pregnancy is a potential teachable moment when the naturally changing health motivations of women could be harnessed for long-term behaviour change [5].

In Ireland, limited data exist on lifestyle behaviours around the time of pregnancy. In contrast, in the United States (US), population based surveillance of maternal behaviours and experiences have been conducted since 1987 through the Centers Disease Control and Prevention (CDC), Pregnancy Risk Assessment Monitoring System (PRAMS) surveillance programme [6]. In 2012, PRAMS Ireland was established to address the lack of population level data on key maternal behaviours and experiences around the time of pregnancy [7].

PRAMS Ireland highlighted the high prevalence of deleterious health behaviours; over 60% of women were not adherent to recommendations during pregnancy such as smoking cessation, alcohol abstinence and folate use [8, 9]. Similarly, the Growing up in Ireland study reported alcohol or smoking exposure during one in four pregnancies [10]. Gestational weight gain (GWG) above the recommended guidelines (Institute of Medicine (IOM)) has been associated with increased risk of complications, and women with a pre-pregnancy Body Mass Index (BMI) > 25 kg/m^2^ are at a higher risk of gaining weight above the guidelines [11]. Current estimates in Ireland indicate that almost half of women have a pre-pregnancy BMI > 25 kg/m^2^ [12]. A longitudinal cohort study also showed that Ireland had higher rates of excess GWG compared to participants in Australia and New Zealand [13]. The benefits of breastfeeding are well documented and recently published literature identified Ireland as having the lowest breastfeeding rates amongst high-income countries [14, 15].

Antenatal care (ANC) involves frequent interaction with pregnant women, providing health care professionals with an ideal opportunity to encourage behaviour change and influence health behaviours [16–18]. During pregnancy, women are more likely to under-estimate the prevalence of unhealthy behaviours and therefore it is important that all women receive advice on healthy behaviours [19]. In Ireland, a general practitioner in primary care and the local maternity hospital provide standard ANC. It is provided free of charge to pregnant women under the Maternity and Infant care scheme and it is recommended that ANC is initiated before 12 weeks [20]. However, despite a high burden of deleterious health behaviours in Ireland, the nature and content of preventive health counselling provided during ANC in Ireland is not well characterized. This study aims to describe the nature, content and quality of preventive health counselling during antenatal care and to compare different risk groups using data from PRAMS Ireland.

## Methods

### Study design

PRAMS Ireland study was established in 2012 in Cork University Maternity Hospital (CUMH) and has been described in detail elsewhere [7, 21]. CUMH is a large urban, obstetric hospital in Ireland, where approximately 9000 live births occur per year (12% of all Irish births). Using hospital discharge records of live births at CUMH from May 14th 2012 to August 18th 2012, a sampling frame of 2424 mother-infant pairs was used to alternately sample 1212 women whom had recently given birth (women were approximately 2–9 months postpartum). Stillbirths and neonatal deaths were excluded. The questionnaire included socio-demographics, health behaviours and experiences before, during and after pregnancy. Eligible participants were sent an invitation letter to the study followed by three surveys, a reminder letter and text message.

### Data

For this study, participants’ age was categorized into four age groups: ≤29, 30–34, 35–39 and ≥ 40 years. Education was classified into those with some third level education (> 14 years) and those with first and second level education only (≤14 years). Women’s ethnic or cultural background was grouped into either “Irish” or “Other” (other white background, African, any other Black background, Chinese or other Asian background). Women were classified as multiparous if they already had delivered one child. Pre-pregnancy BMI was calculated based on self-reported weight before pregnancy in kilograms (kg) and height in meters (m) and categorized as underweight (< 18.5 kg/m^2^), normal (18.5–24.9 kg/m^2^), overweight or obese (> 25 kg/m^2^). Women also reported their weight at the end of pregnancy in kg and this was used to calculate GWG. Unintended pregnancy was defined as a pregnancy that a woman wanted later (mistimed) or did not want at any time (unwanted).

### Preventive health counselling

The World Health Organization state that counselling and education are key components of ANC [22]. To determine the extent of preventive counselling provided, participants were asked: “During any of your antenatal care visits did a doctor, nurse, midwife or other healthcare professional talk with you about any of the things listed below?” Responses included the risks of smoking, alcohol, medication use, illegal drug use, appropriate weight gain, nutrition/diet, breast-feeding and seat-belt use. The quality of ANC in this study was defined as the occurrence of counselling by a health care professional on the topics listed above, as reported by the participants. All health care professionals who come in contact with pregnant women during their pregnancy have the opportunity to provide counselling, these include midwives, pharmacists, public health nurses and doctors. This is one of the quality indicators of ANC to all pregnant women previously reported in the literature [23].

### Maternal behaviours

Positive maternal behaviours were classified according to recommendations from the Institute of Obstetricians and Gynaecologists in Ireland, Royal College of Physicians of Ireland and the Health Service Executive [24]. These included not smoking before or during pregnancy, not consuming alcohol during pregnancy, avoiding excessive weight gain and breastfeeding.

Smoking cessation was defined as use during the 3 months before pregnancy but the absence of use during the last 3 months of pregnancy. Cessation of alcohol was defined as use during the 3 months before pregnancy but the absence of use during the last 3 months of pregnancy. GWG was classified according to IOM guidelines utilizing the self-reported height, pre-pregnancy weight and weight at the end of pregnancy values and converting these to BMI [25]. Breastfeeding was classified as those who initiated breastfeeding and those that did not; women who were recommended not to breastfeed (e.g. health/medication reasons) were excluded from this analysis.

### Analysis

Analyses were conducted using Stata v12. Descriptive analyses were performed to examine the socio-demographics, reported health behaviours (during the 3 months prior to pregnancy and during pregnancy) and the provision of preventive health counselling. Variation in denominators occurred due to incomplete survey data in some sections. Rates of missing data were generally low (< 5%) with the exception of the values required to calculate gestational weight gain, which had 287 missing participants (39.97%).

Univariate and multivariable analyses were conducted to assess whether those women with a need of counselling received counselling for their specific health behaviour, controlling for maternal characteristics (age, marital status, education, ethnicity, parity, health insurance). Missing variables were excluded from multivariable analyses.

## Results

Of the 718 women participating in the study (response rate 61%), 23.3% (*n* = 167) were aged < 29 years, 71.1% (*n* = 510) were aged 30–39, and 5.6% (*n* = 40) were aged ≥40 years. Most women had a third level education (82.3%, *n* = 587), were multiparous (60.0%, *n* = 431) and were white Irish ethnicity (80.7%, *n* = 571). Most pregnancies were planned (80.8%, *n* = 579). The average time of questionnaire completion was 4.6 months post-partum and ranged from two to 9 months.

The prevalence of negative maternal behaviours pre-pregnancy was high. Most women reported alcohol consumption in the 3 months before pregnancy (75.9%, *n* = 545) and almost one in four women were smokers (23.7%, *n* = 170). Over a quarter of the women (28.1%, *n* = 202) were overweight/obese pre-pregnancy and 27.2% (*n* = 195) did not intend to breastfeed (Table 1).

**Table 1:** Characteristic of PRAMS participants stratified by pre-pregnancy health behaviour

		Maternal age				Marital status		Education		Ethnicity		Parity		Insurance		Pregnancy intention	
		≤29	30–34	35–39	≥40	Single / Separated	Married / Cohabiting	Primary / Secondary	Third level	Irish	Non-Irish	0	1+	Private	Public	Yes	No
Total PRAMS	(*n* = 718)	167 (23.3)	286 (39.9)	224 (31.2)	40 (5.6)	57 (8.0)	660 (92.0)	126 (17.7)	587 (82.3)	571 (80.7)	137 (19.4)	287 (40.0)	431 (60.0)	478 (66.9)	237 (33.2)	580 (80.8)	138 (19.2)
Pre-pregnancy health behaviours	Smoking (*n* = 170)	65 (38.5)	67 (39.6)	33 (19.5)	4 (2.4)	20 (11.8)	150 (88.2)	49 (29.0)	120 (71.0)	138 (83.1)	28 (16.9)	82 (48.2)	88 (51.8)	93 (54.7)	77 (45.3)	122 (71.8)	48 (28.2)
	Alcohol (*n* = 545)	116 (21.3)	282 (40.8)	176 (32.4)	30 (5.5)	39 (7.2)	505 (92.8)	88 (16.2)	455 (83.8)	448 (83.1)	91 (16.9)	232 (42.6)	313 (57.4)	385 (70.8)	159 (29.2)	440 (80.7)	105 (19.3)
	Weight > 25 kg/m^2^ (n = 202)	46 (22.8)	77 (38.1)	65 (32.2)	14 (6.9)	16 (7.9)	186 (92.1)	41 (20.6)	158 (79.4)	168 (84.9)	30 (15.2)	81 (40.1)	121 (59.9)	119 (59.5)	81 (40.5)	169 (83.7)	33 (16.3)
	Breastfeeding (*n* = 195)	54 (27.7)	70 (35.9)	62 (31.8)	9 (4.6)	23 (11.8)	172 (88.2)	48 (24.6)	147 (75.4)	185 (96.4)	7 (3.7)	62 (31.8)	133 (68.2)	121 (62.1)	74 (38.0)	150 (76.9)	45 (23.1)

**Fig. 1:**
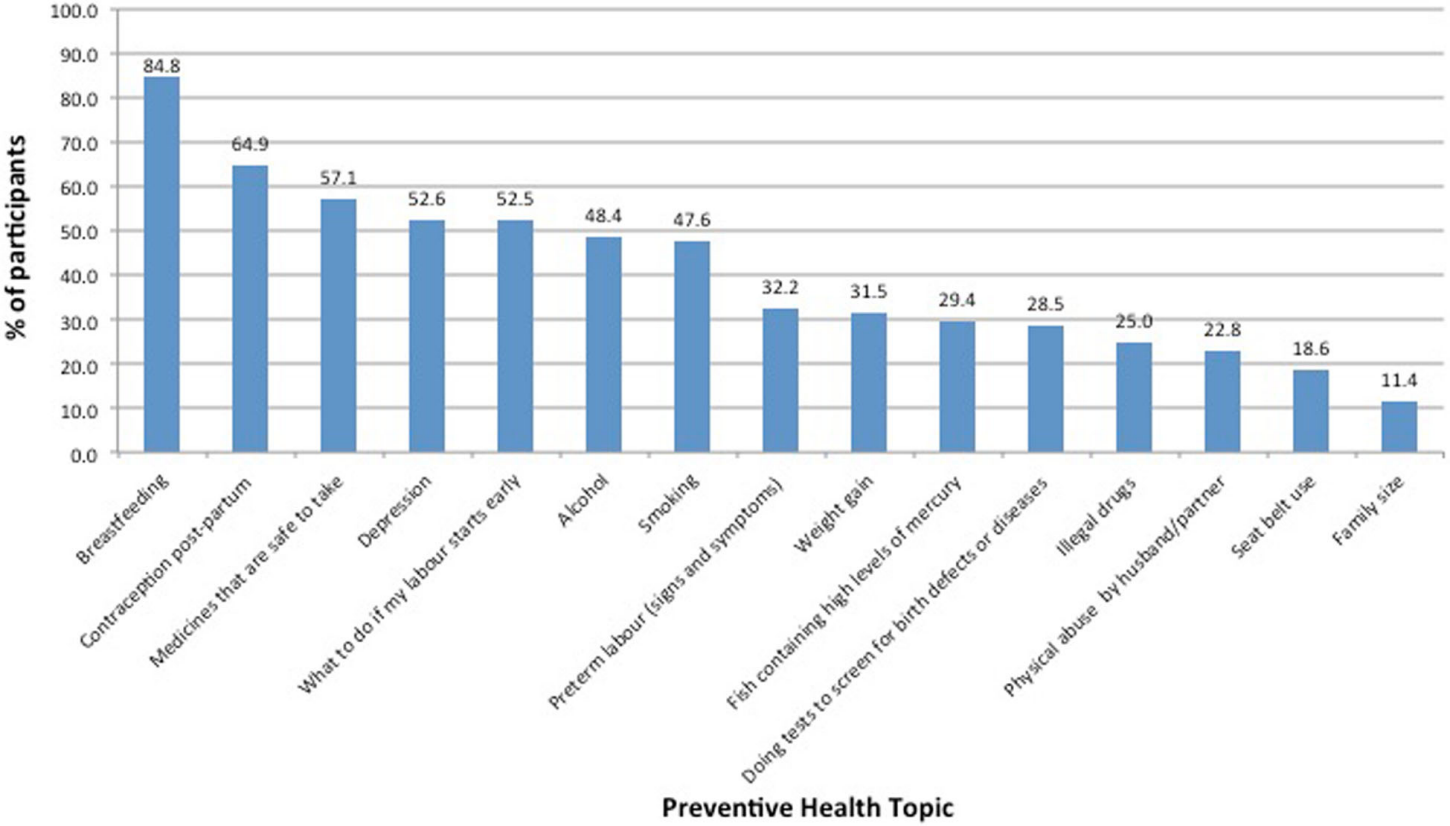
Percentage of participants reporting preventive health counselling by topic

The percentage of women that reported preventive health counselling during antenatal care varied widely (Fig. 1). The highest reported counselling rates were on the topics of breast-feeding (84.8%, *n* = 592, 95% Confidence Interval (CI) 82.2–87.5) and contraception post-partum (64.9%, *n* = 453, 95% CI 61.4–68.4). Participants reported lower rates of counselling on the effects of smoking (47.6%, *n* = 333, 95% CI 43.9–51.3), alcohol (48.4%, *n* = 338, 95% CI 44.7–52.1), appropriate weight gain (31.5%, *n* = 219, 95% CI 28.0–34.9), safe medications (57.1%, *n* = 399, 95%CI 53.4–60.8), illegal drugs (25.0%, *n* = 173, 95%CI 21.7–28.2) and seat belt use (18.6%, *n* = 130, 95%CI 15.7–21.5). The characteristics of those who received counselling are shown in Table 2.

**Table 2:** The socio-demographics of PRAMS participants according to counselling received (smoking, alcohol, weight gain, breastfeeding)

			Maternal age				Marital status		Education		Ethnicity		Parity		Insurance		Pregnancy intention	
Total PRAMS		n = 718	≤29 (n = 167)	30–34 (*n* = 286)	35–39 (*n* = 224)	≥40 (n = 40)	Single/Separated (n = 57)	Married/Cohabiting (*n* = 660)	Primary/Secondary (*n* = 126)	Third level (n = 587)	Irish (n = 571)	Non-Irish (*n* = 137)	0 (*n* = 287)	1+ (n = 431)	Private (*n* = 478)	Public (*n* = 237)	Yes (*n* = 580)	No (*n* = 138)
Counselling received	Smoking	n = 333	110 (65.9)	123 (43.0)	88 (39.3)	12 (30.0)	33 (57.9)	300 (45.5)	77 (61.1)	253 (43.1)	259 (45.4)	72 (52.6)	152 (53.0)	181 (42.0)	193 (40.4)	139 (58.6)	260 (44.8)	73 (52.9)
	Alcohol	n = 338	100 (59.9)	130 (45.5)	96 (42.9)	12 (30.0)	34 (59.6)	304 (46.1)	72 (57.1**)**	263 (44.8)	262 (45.9)	70 (51.1)	156 (54.4)	182 (42.2)	197 (41.2)	139 (58.6)	276 (47.6)	62 (44.9)
	Weight gain	n = 219	55 (32.9)	83 (29.0)	69 (30.8)	12 (30.0)	25 (43.9)	194 (29.4)	36 (28.6)	182 (31.0)	177 (31.0)	39 (28.5)	101 (35.2)	118 (27.4)	143 (29.9)	75 (31.6)	177 (30.5)	42 (30.4)
	Breast feeding	n = 592	142 (85.0)	240 (83.9)	179 (79.9)	30 (75.0)	47 (82.5)	545 (82.6)	101 (80.2)	488 (83.1)	488 (85.5)	97 (70.8)	239 (83.3)	353 (81.9)	396 (82.8)	194 (81.9)	477 (82.2)	115 (83.3)

Note: Percentages are given as a percentage of each sub-type within each demographic

The majority of women adopted healthy behaviours during pregnancy, however a third of pre-pregnancy smokers continued smoking during their pregnancy (31.8%, *n* = 54) and 40% (*n* = 213) of women reported consuming alcohol in the third trimester.

Table 3 shows the factors associated with receiving antenatal counselling on specific health behaviours. Women with private health insurance (29.4% vs 55.3%, AOR 0.54, 95%CI 0.36–0.80) and multiparous women (43.2% vs 56.1%, AOR 0.62, 95%CI 0.45–0.87) were less likely to receive counselling on the effects of alcohol during pregnancy. Smokers were more likely to receive counselling on the effects of smoking (67.7%) compared to non-smokers (39.8%, AOR 2.72, 95%CI 1.84–4.02). Women who continued to smoke during pregnancy were more likely to receive counselling on its effects (83.3% vs 16.4%, OR 5.28 95%CI 2.38–11.74, *p* < 0.05) than women who had ceased smoking. However, women were no more likely to receive counselling on alcohol use whether they reported alcohol use before pregnancy (47.5% vs 51.0%, AOR 0.94, 95%CI 0.64–1.37), during pregnancy (third trimester) (42.1% vs 51.0%, AOR 0.84, 95%CI 0.58–1.22) or discontinued consuming alcohol during pregnancy (50.5% vs 42.7%, AOR 1.19, 95%CI 0.82–1.74). Women with a pre-pregnancy BMI of > 25 kg/m^2^ were no more likely to receive counselling on appropriate weight gain (28.9% vs 31.8%, AOR 0.89, 95% CI 0.56–1.39) than those with a pre-pregnancy BMI of ≤25 kg/m^2^ (Table 3).

**Table 3:** Adjusted odds ratios of receiving antenatal counselling regarding health behaviours

	Smoking counselling AOR (95%CI)	Alcohol use counselling AOR (95% CI)	Weight gain counselling AOR (95% CI)	Breast-feeding counselling AOR (95% CI)
Age (Reference: 26–30)				
*< 30*	1.83 (1.17–2.87)*	1.26 (0.80–1.96)	1.14 (1.58–2.23)*	2.05 (1.00–4.21)
*35–39*	1.02 (0.70–1.48)	0.94 (0.65–1.37)	1.55 (0.94–2.55)	0.64 (0.38–1.07)
*> 40*	0.70 (0.33–1.46)	0.51 (0.24–1.09)	1.50 (0.61–3.68)	0.42 (0.17–0.98)*
Marital status (Reference: Single)				
*Married*	0.97 (0.53–1.78)	0.86 (0.46–1.59)	0.32 (0.14–0.76)*	1.56 (0.66–3.69)
Education (Reference: Primary/Secondary)				
*Third level*	0.76 (0.48–1.20)	0.73 (0.47–1.16)	1.48 (0.75–2.92)	1.04 (0.54–2.01)
Ethnicity (Reference: Non-Irish)				
*Irish*	1.03 (0.67–1.60)	1.04 (0.67–1.62)	1.25 (0.32–4.84)	3.27 (1.92–6.50)*
Parity (Reference: 0)				
*1+*	0.76 (0.55–1.06)	0.62 (0.45–0.87)*	0.69 (0.44–1.08)	1.08 (0.67–1.73)
Insurance (Reference: Public)				
*Private*	0.73 (0.49–1.09)	0.54 (0.36–0.80)*	1.36 (0.76–2.41)	0.72 (0.40–1.29)
Smoking (Reference: Non-smoker)		–	–	–
*3 Months pre-pregnancy*	2.72 (1.84–4.02)*			
*Third trimester*	5.28 (2.38–11.74)*			
*Quit during pregnancy*	0.51 (0.18–1.45)			
Alcohol use (Reference: None)	–		–	–
*3 Months pre-pregnancy*		0.94 (0.64–1.37)		
*Third trimester*		0.84 (0.58–1.22)		
*Quit during pregnancy*		1.19 (0.82–1.74)		
Weight gain (Reference: *GWG within IOM guidelines*) *GWG outside IOM guidelines*	–	–	1.13 (0.72–1.77)	–
Infant feeding (Reference: Breastfeeding) *Stated intent to bottle-feed*	–	–	–	0.74 (0.44–1.26)

*p < 0.05; *AOR* adjusted odds ratio, *CI* confidence interval

## Discussion

This study provides valuable information about the prevalence and quality of preventive health counselling during antenatal care in Ireland. Counselling rates varied widely depending on the health topic and were not always directed at those in most need of the advice.

Breast-feeding counselling rates were similar when compared to US PRAMS data published in 2013 (83% versus 84.8% in this study) [18]. However, counselling rates on all other topics were lower in this study compared to the US where rates were > 70% for many topics. Our results also show that demographic factors and socioeconomic factors influence the prevalence of preventive health counselling. Women under 30 years were significantly more likely to receive counselling on smoking and appropriate weight gain compared to older women. Multiparous women and those with private health insurance were less likely to receive advice regarding alcohol.

It has been shown that counselling regarding weight gain is not seen as a priority for health care providers and is a difficult topic to discuss [26]. Qualitative research highlights that pregnant women would like more advice on appropriate GWG and exercise during pregnancy [27]. Guidelines in Ireland recommend that women with a pre-pregnancy BMI > 25 kg/m^2^ should receive pre-pregnancy dietary counselling to reduce weight prior to conception to minimise the associated risks during pregnancy [24]. However, there is a lack of evidence of the success of such health promotion pre-pregnancy, and often women do not present to health care professionals until after conception [28]. It has also been identified that some women perceive pregnancy as a time to be less rigid with their dietary and activity regimes which suggests that lifestyle counselling during pregnancy could be beneficial [29]. Excess GWG (above the IOM guidelines) is associated with post-partum weight retention long-term which can have life-long health implications for both mother and offspring [30, 31].

The majority of women quit smoking (68.4%) and alcohol (59.8%) during pregnancy in our study. However, despite the known negative effects of smoking, one third of the participants who smoked pre-pregnancy continued to smoke during their pregnancy. The prevalence of alcohol consumption during pregnancy was higher than the prevalence of smoking, despite Irish guidelines recommending abstinence from alcohol throughout pregnancy [32]. Women have reported the lack of clear and consistent advice from health care professionals regarding the use of alcohol [33]. Until recently, recommendations in the United Kingdom suggested that low levels of alcohol were unlikely to be harmful but new guidelines state that there is insufficient evidence to support the safety of any level of alcohol during pregnancy [34, 35]. Recent US guidelines advise abstinence for women planning pregnancy, they also recommend that all women who drink alcohol and are sexually active should be recommended suitable contraception in order to reduce risk of foetal alcohol spectrum disorders [36].

The odds of receiving specific preventive health counselling was not greater for women who reported alcohol use, a pre-pregnancy BMI > 25 kg/m^2^ or those who did not breastfeed. Women who continued to smoke were more likely to receive counselling on its effects, indicating that additional strategies are required. This is in contrast to the US PRAMS data, where smokers and those who consumed alcohol pre-pregnancy were more likely to receive counselling on these specific topics [18].

Counselling rates regarding breastfeeding were found to be high but given the low rates of breast-feeding, counselling needs to be supplemented particularly post-partum. Research has indicated that the most effective interventions are supporting mothers at home and in their communities [37]. Also, there appears to be a cultural barrier in the uptake of breastfeeding. Only seven non-Irish women (5.0%) did not initiate breastfeeding in comparison to 32.4% of Irish women in the study. It has been shown that rates of breastfeeding amongst immigrants to Ireland decline the longer they are resident in Ireland indicating that negative attitudes exist in Irish society towards breastfeeding [38].

Smoking cessation before 15 weeks gestation reduces the risk of low birth weight to those of non-smokers [39]. Providing incentives has been shown to be most successful in smoking cessation and is a promising area of further research [40, 41]. Currently, smoking cessation services are available nationally but specific training on providing counselling during pregnancy is not provided to those who have frequent interaction with pregnant women [42]. A Cochrane review in 2013 found that brief interventions involving opportunistic advice, discussion and encouragement have been shown to increase smoking cessation rates [43].

This is the first study in Ireland to examine the prevalence of preventive health counselling in Ireland. Validated methodology was used based on a CDC surveillance programmed in the US and participants were representative of the Irish birth profile.

The variable prevalence of preventive health counselling during pregnancy in Ireland was demonstrated and also the prevalence of maternal behaviours at two separate time points (before and during pregnancy) showed high rates of negative health behaviours. This information will be used to target future public health initiatives in this area as there is substantial room for improvements.

PRAMS data are self-reported, collected 2–9 months post-partum, and therefore subject to recall bias. The quality of the study is dependent on the ability of participants to recall the counselling they received and their behaviour before and during pregnancy. The under-reporting of alcohol use and smoking during pregnancy due to social desirability bias is also a possibility as women may not want to disclose behaviours known to be harmful. Subjective measurements of weight used in this study are also likely to be under-estimates. A study in a similar population with objective measures of weight pre and post pregnancy found a higher rate of GWG above the IOM guidelines (43%) compared to 35% found in this study [11].

Counselling is a complex process and its effectiveness may be dependent on the experience and training of the provider. No information on who provided the advice during ANC was obtained in this study. Mandatory training in the delivery of counselling during ANC is currently not provided to health care professionals.

There were no data available on the number of antenatal visits of each participant, which may have influenced the receipt of counselling. However, the number of ANC visits has been shown to have no association with improved ANC delivery [44]. It was not possible to classify participants’ need for counselling for other health issues such as exercise due to lack of relevant survey data.

PRAMS in the US has influenced public health policies by providing evidence for the benefits of initiatives in many areas such as folic acid use, pre-conception planning and smoking cessation [45]. Changes to workplace conditions conducive to breastfeeding in the US were shown to increase initiation rates in breastfeeding using data collected from PRAMS [46]. However, behaviour change is a complex process and health promotion of positive behaviours requires a multi-faceted approach. Current guidelines are broad in their focus and do not provide incentives to health care professionals to monitor maternal behaviour during pregnancy. All health care professionals are responsible for providing education, but this has led to non-standardised practises and is likely a cause of the low rates of counselling reported.

There are multiple factors that influence behaviour. This study raises questions about how future initiatives should be delivered when promoting positive maternal behaviours during pregnancy. However, assessing the individual’s need for advice may improve the effectiveness of counselling as shown in other studies [17, 18]. Health promotion advice provided early in the pregnancy or pre-pregnancy has the greatest potential to positively influence maternal behaviours [47, 48]. Pre-pregnancy counselling rates are known to be much lower than during pregnancy [49].

Training and education is also required so that all health care professionals, both in primary and secondary care, provide consistent and tailored messages before and throughout pregnancy to provide a continuum of care. Support services are required post-partum to improve breastfeeding rates. Women who positively change their behaviour should also be followed up post-partum to encourage the continuing benefits of their healthy behaviours such as smoking cessation.

## Conclusion

Pregnancy is an ideal opportunity to encourage positive behaviour change. Preventive health counselling during pregnancy is not routinely provided despite pregnant women reporting potential harmful behaviours. Counselling rates vary widely depending on the health behaviour and patient demographics. This information is vital to target future public health initiatives in this area as there is substantial room for improvements, including risk assessment and standardised counselling before and during ANC. This study suggests that additional strategies are needed to promote positive behaviour before and during the unique opportunity provided by pregnancy.

## Data Availability

The questionnaire and datasets used are available from the corresponding author on request.

## Abbreviations

ANC: Antenatal care
AOR: Adjusted Odds Ratio
BMI: Body Mass Index
CDC: Centers for Disease Control and Prevention
CI: Confidence Interval
CUMH: Cork University Maternity Hospital
GWG: Gestational weight gain
IOM: Institute of Medicine
PRAMS: Pregnancy Risk Assessment Monitoring System
US: United States
